# Generation of Potent and Stable GLP-1 Analogues *via* ‘Serine Ligation’

**DOI:** 10.1021/acschembio.2c00075

**Published:** 2022-03-23

**Authors:** Paul M. Levine, Timothy W. Craven, Xinting Li, Aaron T. Balana, Gregory H. Bird, Marina Godes, Patrick J. Salveson, Patrick W. Erickson, Mila Lamb, Maggie Ahlrichs, Michael Murphy, Cassandra Ogohara, Meerit Y. Said, Loren D. Walensky, Matthew R. Pratt, David Baker

**Affiliations:** 1 Department of Biochemistry and Institute for Protein Design, University of Washington, Seattle, Washington 98195, United States; 2 Department of Chemistry, University of Southern California, Los Angeles, California 90089, United States; 3 Department of Biological Sciences, University of Southern California, Los Angeles, California 90089, United States; 4 Department of Bridge Institute, University of Southern California, Los Angeles, California 90089, United States; 5 Department of Pediatric Oncology and Linde Program in Cancer Chemical Biology, Dana-Farber Cancer Institute; 6 Department of Pediatrics, Harvard Medical School, Boston, Massachusetts 02115, United States

**Keywords:** Bioconjugation, Peptide Therapeutics, Diabetes, Semaglutide

## Abstract

Peptide and protein bioconjugation technologies have revolutionized our ability to site-specifically or chemoselectively install a variety of functional groups for applications in chemical biology and medicine, including enhancing bioavailability. Here, we introduce a site-specific bioconjugation strategy inspired by chemical ligation at serine that relies on a non-canonical amino acid containing a 1-amino-2-hydroxy functional group and a salicylaldehyde ester. More specifically, we harness this technology to generate analogues of glucagon-like peptide-1 (GLP-1) that resemble Semaglutide, a long-lasting blockbuster drug currently used in the clinic to regulate glucose levels in the blood. We identify peptides that are more potent than unmodified peptide and equipotent to Semaglutide in a cell-based activation assay, improve the stability in human serum, and increase glucose disposal efficiency *in vivo*. This approach demonstrates the potential of ‘serine ligation’ for various applications in chemical biology, with a particular focus on generating stabilized peptide therapeutics

## Graphical Abstract

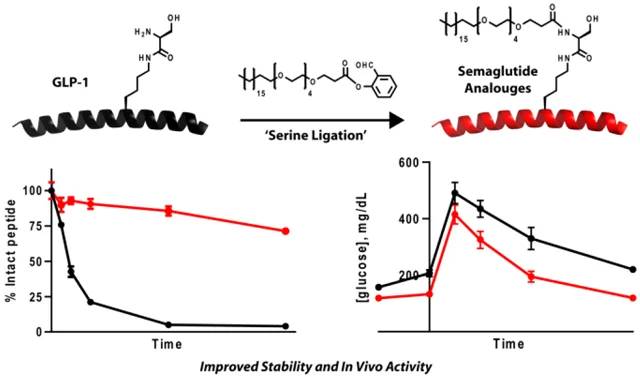

Peptide therapeutics are rapidly becoming approved for clinical use due to their ability to engage their targets with high affinity and specificity.^1^ One well-established class of targets are G protein-coupled receptors (GPCRs), which are activated by peptide hormones.^2^ Although a number of peptide drugs have been FDA approved, such as Teriparatide and Angiotensin II, they often suffer from poor pharmacokinetic profiles *in vivo* that likely arise from proteolytic degradation by endogenous enzymes.^3^ For example, GLP-1(7− 37) has a half-life of only ~2 min due to degradation by dipeptidyl peptidase (DPP-4) cleavage at N-terminal alanine 8.^4^ While there are many strategies aimed at addressing issues with stability, such as incorporation of ‘non-cleavable’ amino acids at the degradation site, they often compromise potency at the expense of stability, including examples of GLP-1.^5,6^ Therefore, new technologies to stabilize peptide therapeutics while maintaining native potency are of great interest.

Peptide and protein bioconjugation has revolutionized our ability to introduce a variety of functional groups for many different applications, including proteomics and high-resolution imaging.^7–9^ Conventional conjugation strategies, such as *N*-Hydroxysuccinimide (NHS) esters, can result in heterogeneous modification due to the presence of multiple sites of reactivity present in peptides or proteins. This polydispersity can engender difficult separation and lead to diminished biological activity. Thus, new methods are needed for versatile site-specific modification. One newer method is Ser/Thr ligation (STL), which is a chemoselective reaction that occurs between a C-terminal salicylaldehyde ester and an N-terminal fragment containing a serine or threonine residue that undergoes reversible imine formation *via* aldehyde capture.^10,11^ Following an acyl shift, a stable *N*,*O*-benzylidene acetal intermediate can be cleaved with acid to liberate a native serine/threonine linkage at the ligation site (Supplementary Figure 1). It is important to note that the *N*,*O*-benzylidene acetal intermediate only forms in the presence of a 1-amino-2-hydroxy functional group, such as N-terminal serine or threonine. Building on this foundation, here we synthesized and incorporated a non-canonical amino acid containing the 1-amino-2-hydroxy functionality to internally and site-specifically modify peptides for various applications in chemical biology, including the generation of potent and stable variants of GLP-1(7− 37) (Figure 1).

To assess the scope and generality of this approach, we first synthesized biotin, cyanine-3, a palmitic acid analogue, and monodisperse poly-ethylene glycol salicylaldehyde esters from commercially available starting materials in one step. All probes were then site-specifically installed onto model peptide **1** containing the 1-amino-2-hydroxy non-canonical amino acid, generating products **2–5** (Scheme 1). To representatively assess product conversion and purity, we monitored the reaction between **1** and biotin salicyaldehyde (Supplementary Figure 2). The reaction proceeds rapidly with near quantitative conversion after 30 min.

Next, we explored how we could harness this bioconjugation strategy to enhance the stability of peptide therapeutics. The most common strategy to extend the half-life of peptide and protein therapeutics is PEGylation and lipidation. In fact, two GLP-1(7– 37) drugs, Semaglutide and Liraglutide, are lipidated and currently used to manage blood glucose for the treatment of type 2 diabetes.^12,13^ Both PEGylation and lipidation provide protection from protease-catalyzed degradation.^14^ Additionally, lipidation promotes binding to circulating human albumin, which releases drugs at a slow, constant rate.^15^

With this in mind, we used STL to synthesize two analogues of GLP-1 that contain a hybrid PEG and fatty acid side-chain resembling Semaglutide (Figure 2). The first peptide (**G1**) was synthesized with an overall yield of 41.3% and was modified at lysine 26, the same position as Semaglutide. As this site is further away from the DPP-4 cleavage site at alanine 8, we also included 2-aminoisobutyric acid (Aib) in place of alanine 8, similar to Semaglutide, to provide additional stability. The main difference between Semaglutide and **G1**, aside from the subtle side-chain modification, is that **G1** maintains the native lysine 34 as conjugation is site-specific with STL. The second peptide (**G2**) was synthesized with an overall yield of 48.1% and was modified at serine 18, as a recent cryo-EM structure of GLP-1R bound to GLP-1 shows serine 18 is solvent exposed and unlikely to perturb any native agonistic function of the peptide hormone.^16,17^ In this particular case, we chose to omit Aib at alanine 8 since the lipid is closer to the N-terminus and likely to shield proteolysis better.

Many biochemical and structural studies have demonstrated that an extended amphipathic α-helix within GLP-1 is responsible for high affinity binding interactions with the extracellular domain of the GLP receptor.^18^ To assess how these modifications might disrupt secondary structure, we used circular dichroism (CD) spectroscopy to observe any changes relative to GLP-1 (Supplementary Figure 2). Relative to GLP-1, which displays a characteristic helical fold, both **G1** and **G2** also show helical structure, however less than native GLP-1. This data is consistent with the lipid modification found on Semaglutide and this loss in structure seems to be induced by the lipid modification.^19^

Endogenous binding of GLP-1 to the GLP-1R results in an intracellular rearrangement that allows recruitment of G-protein, subsequently stimulating the production of cyclic AMP (cAMP) from ATP and leading to glucose-stimulated insulin secretion.^20^ To assess the ability of the lipid modified GLP-1 analogs **G1** and **G2** to activate human GLP-1R, cAMP accumulation was measured in CHO-K1 cells overexpressing the human GLP-1R. Cells were initially treated with native GLP-1 and Semaglutide as reference agonists, which exhibited EC_50_’s of 3.3 ± 0.6 nM and 0.60 ± 0.2 nM (mean ± s.e.m., *n* = 3), respectively (Figure 3a). In comparison, both **G1** and **G2** performed better than unmodified peptide and were roughly equipotent to Semaglutide, with EC_50_’s of 0.97 ± 0.2 nM and 0.73 ± 0.2 nM (mean ± s.e.m., *n* = 3), respectively. These data suggest that neither lipid modification on Lys26 or Ser18 significantly perturbs endogenous function.

To complement our *in vitro* pharmacological profiling of the lipid modified GLP-1 analogues **G1** and **G2**, we next compared the stability of the compounds relative to native GLP-1 and Semaglutide in human serum using reverse-phase high-performance liquid chromatography (RP-HPLC). In this assay, each peptide is incubated in human serum for up to 48 h and aliquots are removed and analyzed by RP-HPLC (Figure 3b and Supplementary Figure 3). As expected, native GLP-1 displayed a relatively short half-life in this assay, t_1/2_ = ~3.5 hr, as the N-terminal Ala8 residue is readily cleaved. In contrast, Semaglutide showed almost no sign of degradation up to 48 h, as its stability is significantly enhanced by the addition of Aib at Ala8 and the lipid modification. Importantly, these half-lives are consistent with previous reports.^11^ Relative to native GLP-1, **G1** displayed a significantly improved stability profile, t_1/2_ = ~40 hr. Although **G1** contains Aib substituted at Ala8 to prevent cleavage by DPP4, these data suggest that other proteases present in human serum can degrade **G1** at other sites. Lastly, **G2** proved to be very stable, with a more than a 14-fold increase in stability relative to native GLP-1, very comparable to Semaglutide.

Given the promising activation and stability data, we next tested our peptides *in vivo* using a standard glucose tolerance test (GTT). More specifically, following a 16h fast, mice were injected intraperitoneally (IP) with 10 nmol/kg peptide followed by 2 g/kg of glucose. For each group, either vehicle (*n* = 8), GLP-1 (*n* = 8), Semaglutide (*n* = 8) or one of the lipidated analogues (*n* = 8 per group), blood-glucose levels were measured using a glucose meter and quantified after different lengths of time (Figure 3c). In this acute GTT, both **G1** and **G2** displayed statistically significant glycemic control compared to unmodified GLP-1 (Supplementary Figure 4), consistent with their *in vitro* data. These data highlight the ability of lipidation to significantly increase stability without compromising potency, thus resulting in improved *in vivo* activity in a mouse model of acute hyperglycemia.

In order to gain molecular insight into how **G1** and **G2** interact with the GLP-1R, we performed computational modeling of the corresponding ligand–receptor complexes, as described in the experimental methods. The GLP-1R peptide binding models were based on the recently published Cryo-EM structure of GLP-1R in complex with unmodified GLP-1 peptide.^16^ The generated models of Semaglutide, **G1**, and **G2** in their complexes with the GLP-1R suggest that lipidation at either serine 18 or lysine 26 are solvent exposed and likely not interfering with any critical contacts responsible for binding or activation (Figure 3d). Additionally, the lipid modification of **G1** is closer to the N-terminus of the peptide, possibly indicating there is an exposed degradation site between the C-terminal Aib residue and the lipid modification.

In conclusion, we introduce a robust site-specific bioconjugation strategy that relies on ‘serine ligation’. A multitude of salicylaldehyde ester probes can be easily synthesized in one step from commercially available carboxylic acids to generate peptides with stable linkages for various applications in chemical biology and medicine. Unlike the chemoselective chemistry that is currently used to install the lipid functional group on Semaglutide, which entails mutation of any other native lysine residue in the native sequence, our site-specific strategy does not require this. This is important as purification of heterogeneously lipidated peptides are extremely difficult to purify as they co-elute. N-terminal serine or threonine residues in peptides can also compete for modification, however this can be avoided by utilizing a simple protecting group strategy, such as allyl serine or N-terminal acetylation. In comparison to other bioconjugation technologies, one main advantage of STL is the native amide linkage formed between the peptide and probe. This may be advantageous in regard to immune response compared to non-native linkages, such as triazoles. Additionally, this method is rapid, metal-free, and orthogonal to other bioconjugation reactions. We applied this technology to produce potent and stable GLP-1 analogues, outfitted with a hybrid PEG and fatty acid side-chain, that resemble the widely used diabetes drug Semaglutide. Both compounds were equipotent to Semaglutide in their ability to activate GLP-1R, displayed significantly improved stability profiles in human serum relative to native GLP-1, and outperformed GLP-1 *in vivo*.

In the future, we aim to utilize this STL bioconjugation strategy to create potent and stable analogues of other GPCRs, such as PTH(1–34), which is clinically marketed by Eli Lilly as Forteo with a half-life of ~5 minutes in the blood.^21^ Additionally, we are considering the possibility of merging STL with amber stop codon technology to scale production by eliminating solid phase peptide synthesis. Like all methods to stabilize peptide drugs (artificial amino acids, polyethylene glycol, etc.), we do not expect STL to be amenable for every target, however this approach demonstrates the potential for creating peptides for an assortment of applications, with a particular emphasis on therapeutic peptides.

## Supplementary Material

SI

## Figures and Tables

**Figure 1.**
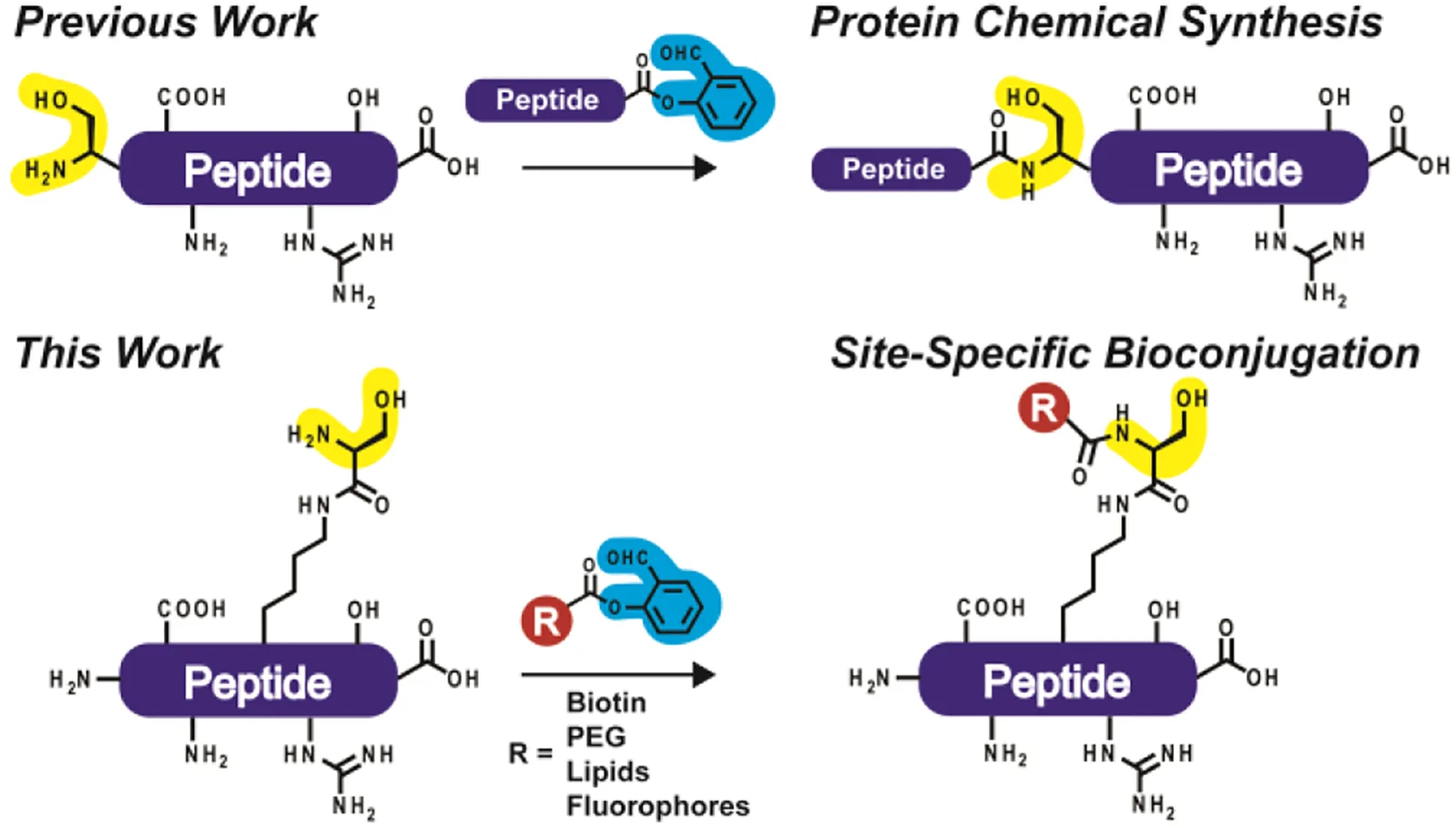
Chemical ligation at serine. (top) Previous efforts have mainly used STL in protein chemical synthesis, semi-synthesis, or peptide cyclization. (bottom) This work relies on a non-canonical amino acid containing the 1-amino-2-hydroxy functionality required for ligation to internally generate site-specific modifications.

**Figure 2.**
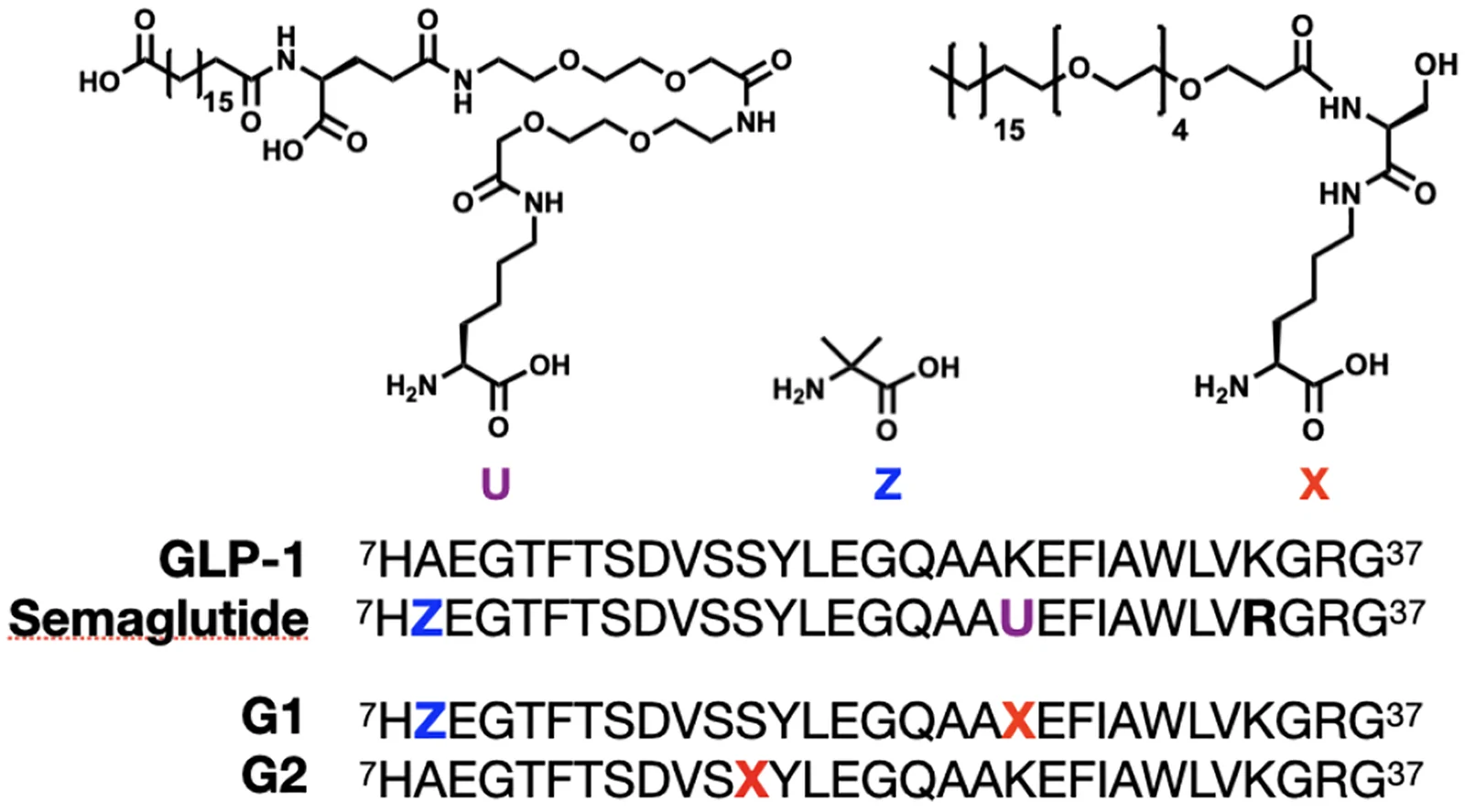
Design of GLP-1 peptide analogues. Primary sequence of GLP-1 and Semaglutide. Peptides **G1** and **G2**, synthesized here *via* STL, contain a C_18_-PEG_4_ modification at position 26 in combination with an Aib residue at position 8 (**G1**) or just a C_18_-PEG_4_ modification at position 18 (**G2**).

**Figure 3.**
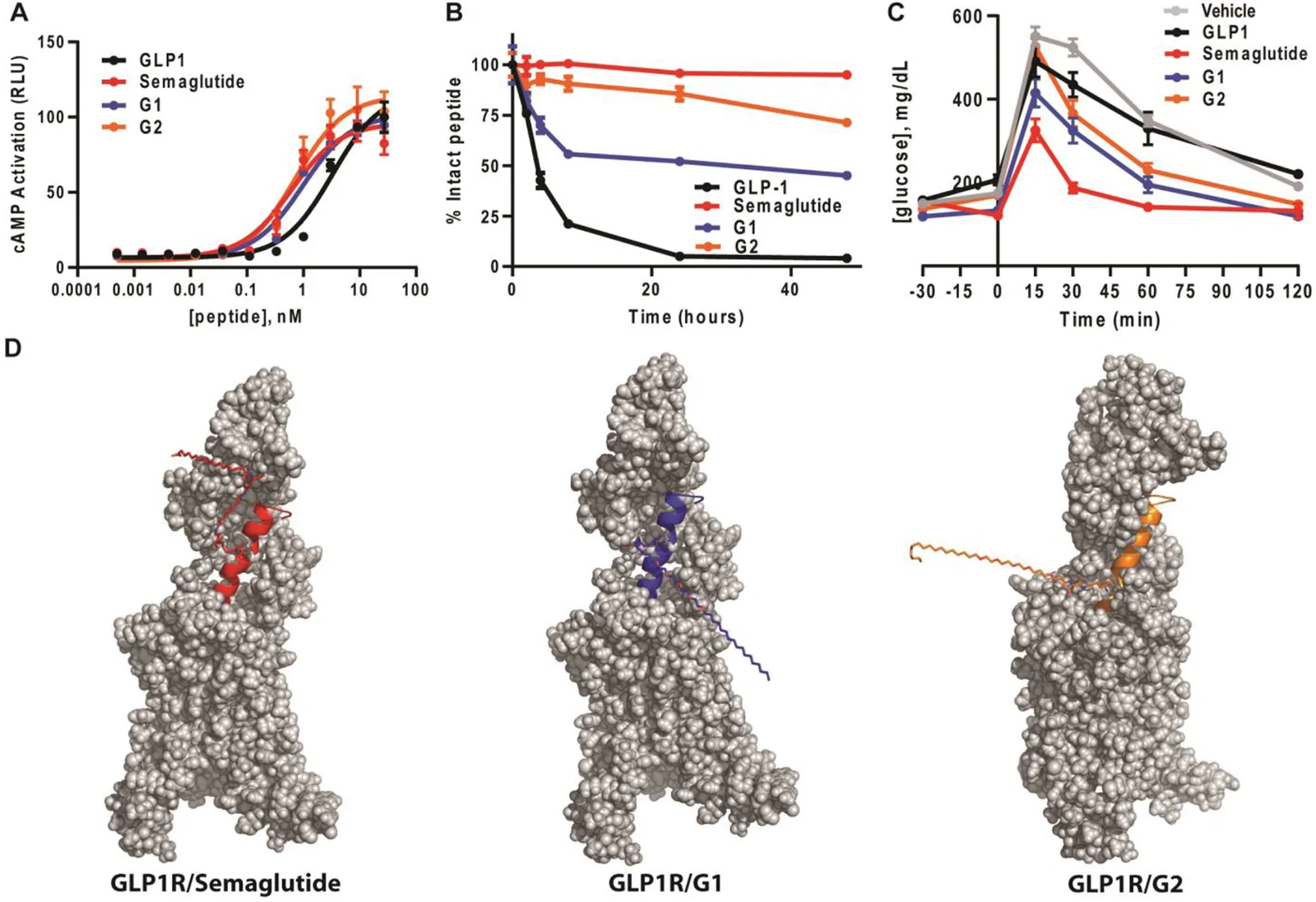
Lipidation does not impact cellular activity, stabilizes GLP-1 from proteolysis, and improves glucose clearance *in vivo*. (a) Lipid alone or with Aib substitution does not affect the EC_50_ of cAMP production when compared to unmodified GLP-1 (*n = 3*). (b) Lipid modification improves the stability of GLP-1. The indicated peptides (*n = 3*) were incubated with human serum for 48 h. The stability of each peptide was measured using RP-HPLC after the indicated lengths of time. (c) Lipidation of GLP-1 improves glucose disposal efficiency in a glucose tolerance test. Lean mice were subjected to a GTT after an overnight fast with 10 nmol/kg IP dosing of vehicle (*n* = 8) or the indicated concentrations of peptide (GLP-1, *n* = 8; Semaglutide, *n* = 8; **G1** and **G2**, *n* = 8) with glucose challenge. Blood glucose levels were then measured after the indicated lengths of time. (d) Models of full length GLP-1R-Semaglutide, GLP-1R-**Gl**, and GLP-1R-**G2** complexes. In all panels, GLP-1R is shown as gray. Semaglutide, **G1**, and **G2** are colored red, blue, and orange, respectively.

**Scheme 1.**
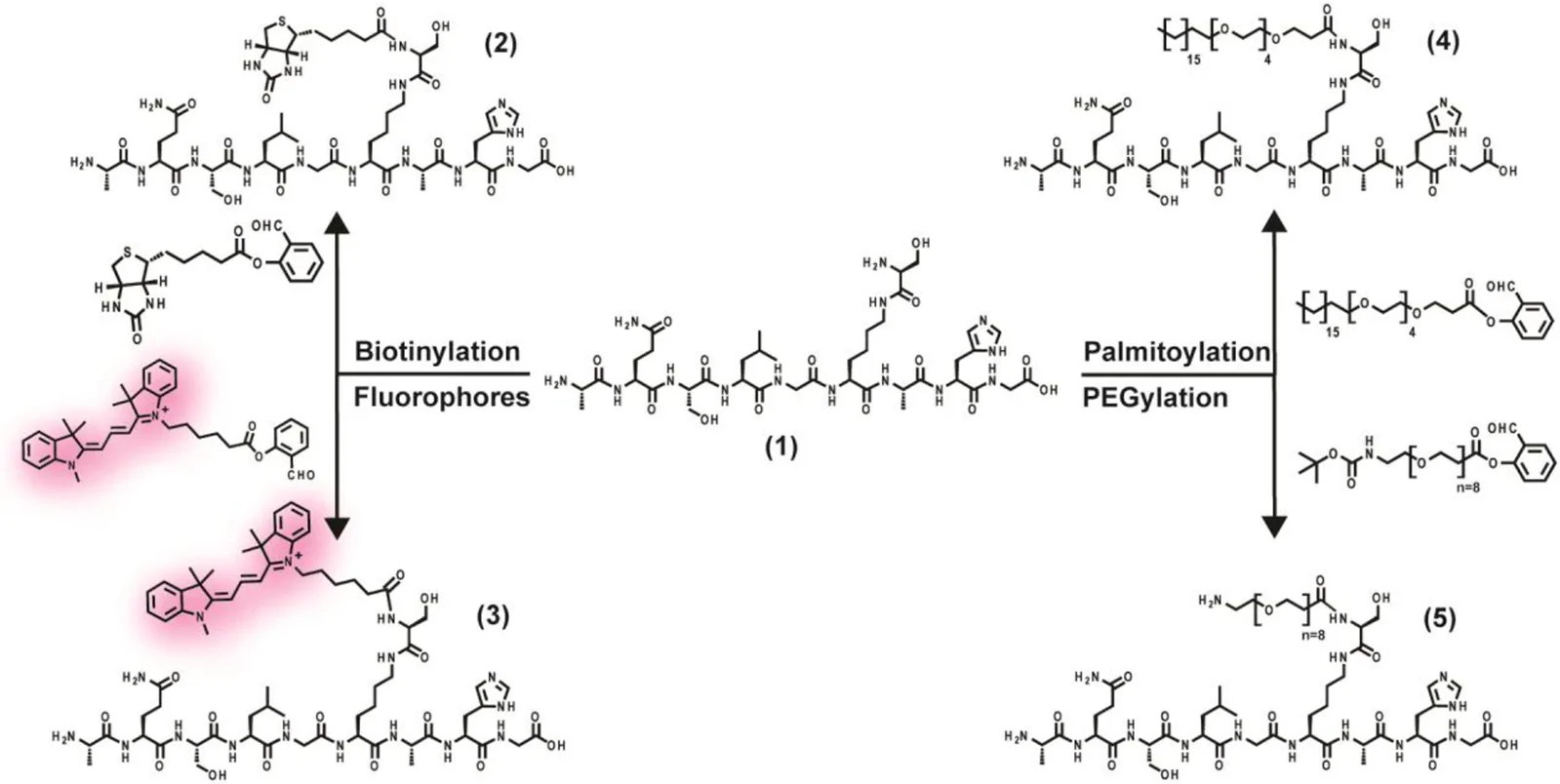
Site-specific modification of an unprotected model peptide *via* STL.^a^ ^*a*^ All reactions were conducted at a concentration of 10 mM in pyridine/acetic acid (1:1 v/v) followed by cleavage using TFA/H_2_O/*i-*Pr_3_SiH (94/5/1, v/v/v).

## ABBREVIATIONS

GPCRs: G protein-coupled receptors
DPP-4: dipeptidyl peptidase
NHS: N-Hydroxysuccinimide
STL: Ser/Thr ligation
Aib: 2-aminoisobutyric acid
GTT: glucose tolerance test
