# Supplementary material for: Generation of Potent and Stable GLP-1 Analogues via ‘Serine Ligation’
Source: ACS Chem Biol. Author manuscript; Available in PMC 2023 Apr 15. (PMC9173702; doi:10.1021/acschembio.2c00075)
Supplement: Supplementary file 1 [file NIHMS1812531-supplement-SI.docx]

Generation of Potent and Stable GLP-1 Analogues *via* ‘Serine Ligation’

Paul M. Levine^1,^*, Timothy W. Craven^1^, Xinting Li^1^, Aaron T. Balana^2^, Gregory H. Bird^5^, Marina Godes^5^, Patrick J. Salveson^1^, Patrick W. Erickson^1^, Mila Lamb^1^, Maggie Ahlrichs^1^, Michael Murphy^1^, Cassandra Ogohara^1^, Meerit Y. Said^1^, Loren D. Walensky^5,6^, Matthew R. Pratt^2,3,4^, and David Baker^1^

^1^Department of Biochemistry and Institute for Protein Design, University of Washington, Seattle, Washington 98195, United States

^2^Departments of Chemistry, ^3^Biological Sciences, and ^4^Bridge Institute, University of Southern California, Los Angeles, California 90089, United States

^5^Department of Pediatric Oncology and Linde Program in Cancer Chemical Biology, Dana-Farber Cancer Institute, and ^6^Department of Pediatrics, Harvard Medical School, Boston, Massachusetts 02115, United States

*Corresponding Author: Paul M Levine, [pmlevine@uw.edu](mailto:pmlevine@uw.edu)

**Supplementary Information**

**Table of Contents:**

**Materials and Methods	Page S2**

**Characterization of Small Molecules, Model Peptide, and GPCR Agonists	Page S5**

**Supplementary Figure 1. Proposed STL Mechanism	Page S10**

**Supplementary Figure 2. Model Reaction conversion	Page S11**

**Supplementary Figure 3. CD Spectroscopy of Peptides	Page S12**

**Supplementary Figure 4. Serum Stability HPLC Traces	Page S12**

**Supplementary Figure 5. AUC of GTT Assay	Page S13**

**References	Page S13**

**Materials:** Biotin was purchased from Sigma Aldrich. Cyanine3 acid was purchased from lumiprobe. C_18_-PEG_4_-COOH and t-boc-N-amido-sPEG_8_-acid were purchased from creative PEGworks and Quanta Biodesign, respectively. Semaglutide acetate was purchased from Bachem. Synthesis of the Lys–Ser dipeptide was conducted by WuXi AppTec as previously reported.^1^ All other reagents were obtained from commercial sources and used without additional purification. All aqueous solutions were prepared using ultrapure laboratory grade water (deionized, filtered, and sterilized) obtained from an in-house ELGA water purification system. Reverse-phase high-performance liquid chromatography (RP-HPLC) was performed using an Agilent Technologies 1260 Series HPLC instrument with a diode array detector. For analytical analysis, a C_18_ reversed-phase HPLC column was used (Higgins). Samples were eluted with a 5-95% acetonitrile/water gradient (0.1% TFA) in 45 minutes with a flow rate of 1 mL/min and monitored at 214 nm. For purifications, semi-preparative C_18_ reversed-phase HPLC columns were used (Higgins). Samples were eluted with a 5-65% or 25-95% acetonitrile/water gradient (0.1% TFA) in 35 minutes with a flow rate of 5.0 mL/min, and monitored at 220 nm. Mass spectra were acquired on an Agilent LC-TOF or Thermo ESI direct inject mass spec.

**Peptide Synthesis:** All peptides were synthesized using standard Fmoc solid-phase chemistry on either 2-Chlorotrityl ProTide (CEM, 0.45 mmol/g) or Rink amide ChemMatrix (PCAS BioMatrix, 0.45 mmol/g) resin using a Liberty Blue peptide synthesizer from CEM. Couplings were performed using DIC (5 equiv, Novabiochem) and Oxyma (10 equiv, Sigma) in DMF followed by Fmoc deprotection with 20% piperidine. All peptides were then cleaved (95:2.5:2.5 TFA/H2O/triisopropylsilane) for 3.5 h at room temperature, precipitated out of cold ether, and purified by reverse-phase HPLC using preparative chromatography. All peptides were characterized by mass analysis using ESI-MS, and the sequence purity was assessed by analytical HPLC.

***Circular Dichroism Spectroscopy:*** Circular dichroism spectra were recorded on a Jasco J-1500 CD spectrometer. Peptides were freshly diluted to a final concentration of 50 μM in 10 mM phosphate buffer (pH 7.4) prior to sample measurement. Spectra were recorded from 250 to 190 nm with a 0.1 nm data pitch, a 50 nm min-1 scanning speed, a 4 sec data integration time, a 1 nm bandwidth, and a 1 mm path length with 3 accumulations, at 25°C. All data were background subtracted from a sample containing only phosphate buffer.

***GLP-1 cAMP Accumulation Assay:*** GLP-1 receptor activation was measured using the cAMP Hunter eXpress assay kit (Eurofins DiscoverX Corporation, #95-0062E2CP2M) in CHO-K1 cells overexpressing the human GLP1R. All reagents were from the assay kit unless stated otherwise. Everything was performed according to the manufacturer’s instructions. All data were analyzed using Prism 8.3.0 (GraphPad Software Inc., San Diego, CA)

***Human Serum Stability Assay:*** Stock solutions of the peptides (10 mM in DMSO) and reconstituted normal human serum (Thermo Scientific) were pre-equilibrated to 37 °C for 15 minutes in separate tubes. For each replicate, peptides and serum were combined to a final concentration of 50 μM and 50% (v/v) respectively, final volume of 150 μL. The reaction tubes were then incubated at 37 °C with 300 rpm shaking in an Eppendorf ThermoMixer. At each time point, 20 μL of the reaction was taken from each replicate and diluted with four-fold excess of cold acetone. The serum proteins were allowed to precipitate for 15 minutes at -20 °C and were removed by centrifugation at 14,000 g, 4 °C for 10 minutes. The supernatant was transferred to a new tube, diluted with the same volume of water, and were stored at -20 °C until HPLC analysis. Chromatographic separations were performed on a C18 reversed phase analytical column (Higgins Analytical, 150x4.6 mm, 5 μm pore size) using a 30-50% B linear gradient over 30 minutes. Solvent A consisted of 0.1% (v/v) TFA in water, and solvent B contained 90% acetonitrile, 0.1% TFA in water. Peak areas corresponding to the intact peptide (as confirmed by MALDI-TOF) were obtained by manual integration using Agilent OpenLab CDS ChemStation.

***In Vivo GTT Test:*** Male B6 mice at 10−12 weeks of age were housed (8 mice/cage) at a constant RT of 24 °C on a normal day-light cycle and provided a standard diet ad libitum. After 1 week of acclimatization, B6 mice (n = 8 per treatment arm) were fasted for 16 h overnight, and the following morning baseline blood glucose levels were measured (OneTouch), followed by intraperitoneal (IP) injection of vehicle (saline) or the indicated peptide (10 nmol/kg). Thirty minutes later, the mice were treated with a bolus IP injection of glucose (2 g/kg, 20% aqueous solution), followed by serial blood glucose monitoring at 0, 15, 30, 60, and 120 min. Samples for the measurement of blood glucose were obtained from the saphenous vein and collected into heparinized capillary tubes. All animal experiments were approved by and performed in accordance with the guidelines and regulations set forth by the Institutional Animal Care and Use Committee of the Dana-Farber Cancer Institute.

***Molecular Modeling:*** Molecular models of the GLP1:GLP1R complex (PDBid: 6X18) were modified at the amino-acid level to reflect the different design sequences. Each structure was minimized using the Yasara software package; Amber14 force-field steepest descent minimization.

**Synthesis of Salicylaldehyde Esters and Ligation Reaction Conditions**

*Synthesis of Salicylaldehyde Esters:* Biotin, Cyanine3, C_18_-PEG_4_-COOH, and t-boc-N-amido-sPEG_8_-acid were added to salicylaldehyde (1.1 eq.), DIC (1.2 eq), and DMAP (0.1 eq) in dry DCM (2-4 mL). Following completion of the reaction, DCM was removed via rotavap and the crude product resuspended in water/acetonitrile and purified by semi-preparative HPLC. Products were characterized by NMR using DMSO-D6.

*General Procedure for Ligation to Model Peptide:* Salicylaldehyde esters (1 equiv.) and the peptide fragment (1.0 equiv.) were dissolved in pyridine/acetic acid (1:1 v/v) to a final concentration of ~0.010-0.05 M. The reaction(s) were stirred at room temperature and monitored using LC/MS. Following completion of the reaction, the solvent was removed by lyophilization and the intermediate product was treated with TFA/H_2_O/*i-*Pr_3_SiH (94/5/1, v/v/v) for 10 min (2 hours for PEG with Boc) to give the product containing a native amide bond at the ligation site.

*General Procedure for Ligation to GLP-1 (7-37):* GLP-1 (10 mg) was dissolved in pyridine/acetic acid (1:1 v/v) to a final concentration of 10 mM and corresponding salicylaldehyde ester (1 equiv.) was added. The reaction was stirred at room temperature and monitored by HPLC. Following completion, the solvent was removed by lyophilization and the intermediate was treated with TFA/H_2_O/*i-*Pr_3_SiH (94/5/1, v/v/v) for 15 min, and 2 hr for Boc protected PEG, to give the product containing a native amide bond at the ligation site.

**Characterization of small molecules, model peptide, and GPCR agonists**

^1^H NMR (500 MHz, DMSO-d6), δ10.09 (1H, s), δ7.89 (1H, dd J =7.9, 1.7 Hz ), δ7.76 (1H, dt, J = 7.6, 1.9 Hz), δ7.48 (1H, app t, J = 7.56), δ7.29 (1H, dd, J = 8.17, 0.87 Hz), δ3.789 (2H, t, J = 6.36), δ3.5 (14H, m), δ3.35 (2H, t, J = 6.57 Hz), δ2.92 (2H, t, J = 6.27), δ1.45 (2H, m), δ1.23 (30H, br s), δ0.85 (3H, t, J = 6.78 Hz),

^1^H NMR (500 MHz, DMSO-d6), δ10.06 (1H, s), δ7.92 (1H, app d, *J* = 7.64 Hz), δ7.76 (1H, app t, *J* = 7.74 Hz), δ7.50 (1H, app t, *J* = 7.55 Hz), δ7.30 (1H, app d, *J* = 8.62 Hz), δ6.5-6.3 (3H, br), δ4.31 (1H, s), δ4.16 (2H, s), δ3.15 (2H, br s), δ2.88 (2H, m), δ2.67 (3H, m), δ2.59 (2H, app d, *J* = 12.7 Hz), δ1.7 (4H, br s), δ1.54-1.45 (5H, m)

^1^H NMR (500 MHz, DMSO-d6), δ10.04 (1H, s), δ8.35 (1H, t, *J* = 13.8 Hz), δ7.9(1H, dd, *J* = 7.77, 1.45 Hz), δ7.75 (1H, td, *J* = 7.92, 1.54 Hz), δ7.64 (2H, dd, *J* = 7.49, 2.56 Hz), δ7.51-7.43 (6H, m), δ7.33-7.28 (3H, m), δ7.23 (1H, d, *J* = 8.18 Hz), δ6.48 (3H, dd, *J* = 24.0, 13.5 Hz), δ4.15 (3H, app t, *J* = 7.42 Hz), δ 3.64 (3H, s), δ2.71 (2H, t, *J* = 7.57 Hz), δ1.84-1.72 (5H, m), δ1.69-1.68 (15H, m), δ1.57-1.51 (2H, m)

^1^H NMR (500 MHz, DMSO-d6), δ10.09 (1H, s), δ7.9-7.88 (1H, app d, *J* = 7.83 Hz), δ7.78-7.75 (1H, app t, *J* = 8.05 Hz), δ7.5-7.47 (1H, t, *J* = 7.79 Hz), δ7.3-7.29 (1H, d, *J* = 8.3 Hz), δ6.74 (1H, app t, *J* = 5.6 Hz), δ3.79 (2H, t, *J* = 6.47 Hz), δ7.5-7.47 (1H, t, *J* = 7.79 Hz), δ3.57-3.49 (21H, m), δ3.37-3.35 (8H, m), δ3.07-3.03 (2H, q, *J* = 5.92 Hz), δ2.92 (2H, t, *J* = 6.39 Hz), δ1.37 (9H, s)

**Figure S1.** Proposed ligation mechanism to form native Ser/Thr linkages.

**Figure S2.** Model reaction between peptide 1 and biotin salicyaldehyde. Bottom trace is 10mM peptide 1 in 50:50 pyridine/acetic acid. Upper trace is reaction after 30 min incubation with biotin salicyaldehyde. * denotes unknown impurities from the reaction.

**Figure S3. Secondary structure of peptides in solution.** Circular dichroism (CD) spectra were collected for freshly dissolved samples in 10 mM phosphate buffer (pH= 7.4) of the indicated peptides at 50 μM concentration. Semaglutide was not soluble as a 5 mM stock in 10% acetonitrile.

**Figure S4. Lipidation improves the serum stability of GLP-1.** The indicated peptides were incubated in triplicate with human serum for 48 h. Representative RP-HPLC traces that were used for quantitation are shown.

**Figure S5.** Blood glucose area under the curve (AUC) calculated from the data in Figure 3C. Error bars represent ± s.e.m., and statistical significance of differences in AUC from GLP-1 was calculated using one-way ANOVA followed by Dunnett’s post-test.

**References**

1) C. H. P. Cheung, J. Xu, C. L. Lee, Y. Zhang, R. Wei, D. Bierer, X. Hunag, and X. Li, *Chem. Sci*., 2021, **12**, 7091.
